# New therapeutic targets in rare genetic skeletal diseases

**DOI:** 10.1517/21678707.2015.1083853

**Published:** 2015-09-24

**Authors:** Michael D Briggs, Peter A Bell, Michael J Wright, Katarzyna A Pirog

**Affiliations:** ^a^ Newcastle University, Institute of Genetic Medicine, International Centre for Life, Central Parkway, Newcastle-upon-Tyne, NE1 3BZ, UK; ^b^ Newcastle University, Institute of Genetic Medicine, International Centre for Life, Newcastle-upon-Tyne, NE1 3BZ, UK

**Keywords:** achondroplasia, cartilage, cell signalling, endoplasmic reticulum stress, genetic skeletal disease, multiple epiphyseal dysplasia, pseudoachondroplasia, skeletal dysplasia

## Abstract

***Introduction:*** Genetic skeletal diseases (GSDs) are a diverse and complex group of rare genetic conditions that affect the development and homeostasis of the skeleton. Although individually rare, as a group of related diseases, GSDs have an overall prevalence of at least 1 per 4,000 children. There are currently very few specific therapeutic interventions to prevent, halt or modify skeletal disease progression and therefore the generation of new and effective treatments requires novel and innovative research that can identify tractable therapeutic targets and biomarkers of these diseases.

***Areas covered:*** Remarkable progress has been made in identifying the genetic basis of the majority of GSDs and in developing relevant model systems that have delivered new knowledge on disease mechanisms and are now starting to identify novel therapeutic targets. This review will provide an overview of disease mechanisms that are shared amongst groups of different GSDs and describe potential therapeutic approaches that are under investigation.

***Expert opinion:*** The extensive clinical variability and genetic heterogeneity of GSDs renders this broad group of rare diseases a bench to bedside challenge. However, the evolving hypothesis that clinically different diseases might share common disease mechanisms is a powerful concept that will generate critical mass for the identification and validation of novel therapeutic targets and biomarkers.

## 1. Introduction

Genetic skeletal diseases (GSDs) are an extremely diverse and complex group of rare genetic conditions that primarily affect the development and homeostasis of the osseous skeleton [1,2]. Although individually rare, as a group of related genetic diseases, GSDs have an overall prevalence of at least 1 per 4,000 children, which extrapolates to a minimum of 225,000 people in the European Union. This burden in pain and disability leads to poor quality of life and high healthcare costs.

There are more than 450 unique and well-characterized phenotypes that range in severity from relatively mild to severe and lethal forms and are described in detail in the 2011 Nosology and Classifications of the GSDs [1]. Forty different diagnostic groups have been recognized to date, which are defined by a combination of molecular, biochemical and/or radiographic criteria. The 2011 Nosology includes 316 conditions associated with one or more of 226 different genes; however, the continued genetic and molecular characterization of GDSs has led to a better defined clinical-molecular classification and a greater understanding of their aetiology [2]. The generation and in-depth analysis of relevant cell and animal models has also increased our understanding of disease mechanisms and has identified phenotype-specific disease signatures through ‘omics’-based analysis.

GSDs are difficult human diseases to treat, particularly when the pathological process begins before birth and can affect the entire skeletal system. Furthermore, since it is now known that the skeleton has close physiological relationships with many other tissue systems in the body, and mutant genes may have pleiotropic effects, patients affected by GSDs may also have serious complications with other organs, including the peripheral nervous system, brain, bone marrow, immune system, pancreas, kidney, heart, muscle and tendon.

The translation of state-of-the-art technology into quantifiable patient benefits such as the development of new treatments or effective biomarkers has been extremely limited for GSDs. The few notable exceptions include Biomarin’s drug candidate for achondroplasia (ACH), a C-type natriuretic peptide (CNP) analogue PG-CNP37 (BMN-111) [3], bisphosphonate treatment for OI [4] and fibrous dysplasia [5], enzyme replacement in lysosomal storage diseases [6] or hematopoietic stem cell transplantation for infantile osteopetrosis [7]. This review will explore a select range of GSDs and propose shared disease mechanisms that hold the promise as potential therapeutic targets.

## 2. Genetic mouse models provide new insight into shared disease mechanisms

Over the last 20 years, the generation and in-depth analysis of transgenic, knock-out, knock-in and ENU-derived mice models of GSDs have generated valuable knowledge of disease mechanisms *in vivo*. Several recent reviews have highlighted both strengths and weaknesses of various modelling approaches [8]; however, these methodologies are still the gold standard for generating relevant *in vivo* models to investigate skeletal pathobiology. These will also act as pre-clinical models when new therapeutic targets are identified and validated.

## 3. ER stress is a shared mechanism and therapeutic target in a range of GSDs resulting from dominant-negative mutations in cartilage structural proteins

The extracellular matrix (ECM) of cartilage is a highly organized composite material comprising numerous structural macromolecules such as collagens (Types II, IX, X and XI), proteoglycans (aggrecan) and glycoproteins (matrilin-3 and cartilage oligomeric matrix protein [COMP]). Mutations have now been identified in all the genes encoding the major structural components of the cartilage ECM and result in a diverse group of both dominant and recessive GSDs. These assorted mutations fall into two broad classes: qualitative mutations, such as those that have dominant-negative (antimorphic) effects, and quantitative mutations that result in haploinsufficiency and/or a complete loss of protein function. This section will focus specifically on dominant-negative (antimorphic) mutations, which affect conserved residues that are structurally and functionally important for normal protein folding and function (Table 1).

**Table 1.** **Disease mechanisms and potential therapeutic targets in selected GSDs resulting from antimorphic mutations in cartilage structural proteins.**

**Gene**	**Protein**	**Disease**	**In**	**Molecular mechanism**	**Cell and/or tissue mechanism**	**Target (s)**	**Treatment**	**Ref.**
*COL2A1*	Type II collagen	Various type II collagenopathies (for details see legend)	AD	Various antimorphic missense mutations and small in-frame deletions	Some mutations cause ER stress, reduced chondrocyte proliferation and increased apoptosis Disrupted ECM organization	ER stress through pharmacological intervention. Mutant protein degradation by the proteasome or autophagy	TMAO	[11–13]
*COL9A1*	Type IX collagen	MED (EDM2, 3 & 6)	AD	Exon skipping and in-frame deletion in COL3 domain	Potential disruption to collagen fibril structure and cartilage ECM composition organization	No known target	None tested	
*COL9A2*								
*COL9A3*								
*COL10A1*	Type X collagen	Metaphyseal chondrodysplasia, Schmid type	AD	Various antimorphic missense mutations and small in-frame deletions	ER stress, UPR and chondrocyte reprogramming Disrupted ECM organization	ER stress through pharmacological intervention. Mutant protein degradation by the proteasome or autophagy	None tested	[10,16,21]
*COL11A1*	Type XI collagen	OSMED	AR	Homozygous missense mutations	Some mutations may cause ER stress, reduced chondrocyte proliferation and increased apoptosis Disrupted ECM organization	ER stress through pharmacological intervention. Mutant protein degradation by the proteasome or autophagy	None tested	
*COL11A1*		Stickler syndrome type 2	AD	Heterozygous missense mutations			None tested	
*COL11A1*		Marshall	AD	Exon skip resulting in an in-frame deletion and missense mutations			None tested	
*COL11A2*		Stickler syndrome type 3	AD	Exon skip resulting in an in-frame deletion			None tested	
*COL11A2*		OSMED/WZS	AD	Heterozygous missense mutations			None tested	
*COL11A2*		Fibrochondrogenesis 2	AR	Homozygous mutations predicted to result in-frame deletions in triple helix			None tested	
*COMP*	COMP	Pseudoachondroplasia MED	AD	Various antimorphic missense mutations and small in-frame deletions	ER stress, reduced chondrocyte proliferation and increased/dysregulated apoptosis Disrupted ECM organization	ER and/or oxidative stress through pharmacological intervention. Mutant protein degradation by the proteasome or autophagy	Aspirin Lithium Valproate SPB	[18–20,26–29]
*MATN3*	Matrilin-3	MED (EDM5)	AD	Various antimorphic missense mutations and small in-frame deletions	ER stress, UPR, reduced chondrocyte proliferation and dysregulated apoptosis Disrupted ECM organization	ER stress through pharmacological intervention. Mutant protein degradation by the proteasome or autophagy	SPB	[17,22,23]
*ACAN*	Aggrecan	Idiopathic short stature	AD	L2355P antimorphic missense mutation	Mutant aggrecan appears to be secreted. Possible altered cartilage ECM composition though disrupted binding to ECM components via the aggrecan G3 domain	No known target	None tested	
		Spondyloepimetaphyseal dysplasia	AR	D2267N antimorphic missense mutation				
		Osteochondritis dissecans	AD	V2303M antimorphic missense mutation				

Type II collagenopathies include: Achondrogenesis, type II or hypochondrogenesis (200610), avascular necrosis of the femoral head (608805), Czech dysplasia (609162), epiphyseal dysplasia, multiple, with myopia and deafness (132450), Kniest dysplasia (156550) Legg-Calve-Perthes disease (150600), osteoarthritis with mild chondrodysplasia (604864), otospondylomegaepiphyseal dysplasia (215150), platyspondylic skeletal dysplasia, Torrance type (151210), SED congenital (183900), SED, Namaqualand type, SMED Strudwick type (184250), spondyloperipheral dysplasia (271700). AD: Autosomal dominant; AR: Autosomal recessive; COL: Collagenous domain; ECM: Extracellular matrix; ER: Endoplasmic reticulum; G3: Globular domain; GSDs: Genetic skeletal diseases; MED: Multiple epiphyseal dysplasia; SPB: Sodium phenylbutyrate; TMAO: Trimethylamine N-oxide; UPR: Unfolded protein response.

The endoplasmic reticulum (ER) is a distinct organelle of eukaryotic cells and plays the major role in the synthesis, folding and trafficking of proteins entering the secretory pathway. The ER has a highly sophisticated quality control mechanism for ensuring that misfolded mutant proteins do not accumulate in, or enter the secretory pathway. Eukaryotic cells have a homeostatic mechanism for maintaining the protein-folding equilibrium of the ER, which is known as the unfolded protein response (UPR) [9]. However, the UPR has evolved to resolve short-term acute ER stress such as high protein load, heat shock or ischemia, but not prolonged ER stresses due to rare events such as the misfolding of mutant proteins in human genetic diseases [10]. A prolonged UPR can eventually have detrimental effects on chondrocyte phenotype, differentiation and viability [10].

An extensive allelic series of glycine and non-glycine substitutions in type II collagen have been introduced into mice to model a diverse range of type II collagenopathies [11,12]. A common feature in many of these mouse models was the evidence of ER stress, translating in some cases into a reduction in chondrocyte proliferation and an increase in apoptosis. Moreover, the recent development of induced pluripotent stem (iPS) cells, and their differentiation into relevant cell types such as chondrocytes (iChon), has allowed the in-depth analysis of cells from patients with various type II collagenopathies and the testing of potential corrector molecules such as trimethylamine *N*-oxide (TMAO) [13], which is a chemical chaperone that can alleviate mutant protein aggregation and ER stress [14,15]. These studies have provided preliminary proof-of-principle evidence that molecular chaperones may serve as therapeutic drug candidates [13].

**Figure 1.**
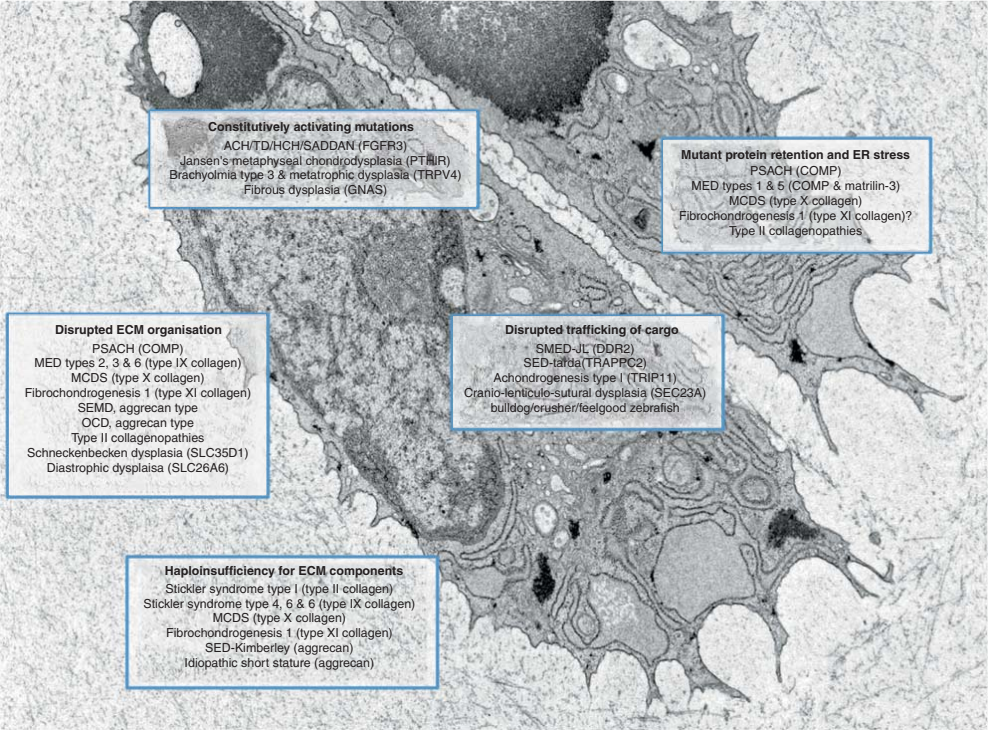
**Schematic showing chondrocytes and pericellular cartilage matrix from the growth plate of a 1-week-old wild type mouse.** Five fundamental disease mechanisms are highlighted along with a selection of associated genetic skeletal diseases.

The role of ER stress in GSDs might best be exemplified by mutations in COMP, matrilin-3 and type X collagen resulting in pseudoachondroplasia (PSACH), multiple epiphyseal dysplasia (MED) [8] and metaphyseal chondrodysplasia type Schmid (MCDS), respectively (Figure 1 & Table 1) [16]. Over the last 10 years, the extensive analysis of mouse models for the *MATN3* (V194D) [17], *COMP* (D469del, T585M) [18,19] and *COL10A1* (N617K) [16] mutations has been performed, which has allowed a direct comparison of disease mechanisms [8,20]. Furthermore, the application of ‘omics’-based investigations (mRNA and protein) has allowed genotype-specific disease signatures to be derived and either shared or discrete downstream genetic pathways to be identified [8,18,21,22].

Disease Key: ACH: Achondroplasia; TD: Thanatophoric dysplasia; HCH: Hypochondroplasia; SADDAN: Severe achondroplasia with developmental delay and acanthosis nigricans; PSACH: Pseudoachondroplasia; MED: Multiple epiphyseal dysplasia; SMED-JL: Spondylo-meta-epiphyseal dysplasia short limb-hand type; SED: Spondyloepiphyseal dysplasia; MCDS: Metaphyseal chondrodysplasia, Schmid type; SEMD: Spondyloepimetaphyseal dysplasia; OCD: Osteochondritis dissecans. Gene Key: FGFR3: Fibroblast growth factor receptor 3; PTH1R: Parathyroid hormone 1 receptor; TRPV4: Transient receptor potential cation channel subfamily V member 4; GNAS: Guanine nucleotide binding protein, alpha stimulating; COMP: Cartilage oligomeric matrix protein; DDR2: Discordin domain receptor 2; TRAPPC2: Trafficking Protein Particle Complex 2; TRIP11: Thyroid Hormone Receptor Interactor 11; SEC23A: Sec23 homolog A.

Interestingly, both *Matn3* (V194D) and *Col10a1* (N617K) mutations cause misfolding and retention of the relevant mutant protein, inducing ER stress and a classical UPR, primarily characterized by the up-regulation of ER chaperones BiP, Grp94 and a range of protein disulphide isomerases (PDIA) [21,22]. Hartley and colleagues [23] commented on a similar increase in specific PDIAs (PDIA1, 3, 4 and 6) in chondrocytes from *Col10a1* ^N617K^ and *Matn3* ^V194D^ mutant mice and also noted that two novel ER stress-related genes, *Armet* and *Creld2*, were also significantly upregulated in these two models [23]. The cartilage-specific knock-out of both *Armet* and *Creld2* has resulted in mice with growth plate dysplasia, thus confirming their important role in skeletal development (our unpublished observations). Moreover, the recent cartilage-specific knock-out of PDIA3 (also called ERP57/GRP58) caused ER stress resulting in reduced proliferation and accelerated apoptotic cell death of chondrocytes in the growth plate [24]. Finally, the cartilage-specific ablation of an entire UPR branch (i.e. Xbp-1 signalling) also resulted in a chondrodysplasia that was characterized by reduced chondrocyte proliferation and leading to delayed cartilage maturation and mineralization [25].

In contrast, the accumulation of mutant COMP has been demonstrated to result in the induction of novel stress pathways, which are characterized by changes in the expression of groups of genes implicated in oxidative stress (ER dependent), cell cycle regulation and apoptosis [18,26,27]. In this context, Posey and colleagues have recently demonstrated that the postnatal administration of aspirin to a transgenic dox-induced COMP-overexpression model of PSACH abolished mutant COMP intracellular retention and had beneficial effects on chondrocyte proliferation, apoptosis and final bone length [28]. However, this study failed to show increased secretion of wild type or mutant COMP upon treatment and also to identify a mechanism by which aspirin may reduce mutant COMP retention and modulate chondrocyte phenotype and bone growth in PSACH [28]. Nevertheless, these are interesting findings that require further validation.

In summary, these recent studies using a complimentary group of genetically relevant mouse models and cartilage-specific knock outs have demonstrated the key role that ER stress plays in the initiation and progression of growth plate dysplasia and reduced bone growth (Figure 1). Moreover, preliminary studies suggest that ER stress is a good therapeutic target that can be influenced through small molecule intervention, and to date, the use of trimethylamine *N*-oxide (TMAO) [13], lithium, valproate [29], sodium phenylbutyrate (SPB) [22,29] and various antioxidant and anti-inflammatory agents [28] have been tested in both cell and mouse models with varying degrees of success.

## 4. Disruption to protein trafficking in chondrocytes leads to various chondrodysplasia phenotypes through different mechanisms

Missense mutations in the discordin domain receptor 2 (DDR2) have been shown to cause the rare autosomal recessive spondyo-meta-epiphyseal dysplasia with short limbs and abnormal calcifications (SMED-SL) due to either protein trafficking defects or loss of ligand binding [30]. DDR2 is a plasma membrane receptor tyrosine kinase that functions as a collagen receptor and missense mutations in DDR2 result in either its retention within the ER, or loss of collagen-binding activity and transmembrane signalling. Both mechanisms most likely lead to retention in the ER of the DDR2 cargo (e.g. fibrillar collagens), thereby further exacerbating ER stress due to mutant DDR2 alone [30]. The deletion of DDR2 in mice has previously been reported to cause reduced chondrocyte proliferation and dwarfism [31], suggesting a role for DDR2 in cell-matrix attachment and in pathological conditions where increased cell proliferation and ECM turnover are coupled (Table 2) [32].

**Table 2.** **Disease mechanisms and potential therapeutic targets in selected GSDs resulting from a disruption to protein trafficking in chondrocytes.**

**Gene**	**Protein**	**Disease**	**In**	**Molecular mechanisms**	**Cell and/or tissue mechanism**	**Target(s)**	**Ref.**
*DDR2*	Discordin domain receptor 2	Spondyo-meta-epiphyseal dysplasia with short limbs	AR	Missense and exon skipping mutations	Retention within the ER, loss of collagen-binding activity and signalling. ER stress, reduced chondrocyte proliferation	Modulation of the secretory pathway. Restoration of normal rates of secretory protein synthesis and secretion	[30–32]
*TRAPPC2*	Trafficking Protein Particle Complex 2	Spondyloepiphyseal dysplasia Tarda	XR	Various antimorphic and loss of function (nonsense) mutations	Defect in the trafficking and secretion cartilage structural proteins		[33–35]
*TRIP11*	Thyroid Hormone Receptor Interactor 11	Achondrogenesis type I	AR	Homozygous or compound heterozygous for loss-of-function mutations	Disrupted golgi structure, ER stress, abnormal chondrocyte differentiation and increased apoptosis		[39]
*SEC23A*	Protein transport proteins	Cranio-lenticulo-sutural dysplasia	AR	Homozygous missense mutation causes loss of protein function	Accumulation of proteins, in particular fibrillar collagens and matrilins, within the ER of relevant cell types		[36]
*Sec23a*		Zebrafish *crusher* mutant	AR	Homozygous nonsense mutation causes loss of protein function			[37]
*Sec24d*		Zebrafish *bulldog* mutant	AR	Loss of protein function			[38]

AD: Autosomal dominant; AR: Autosomal recessive; ER: Endoplasmic reticulum; GSDs: Genetic skeletal diseases; XR: X-linked recessive.

Similarly, the ER export of procollagen is also controlled in part by the ubiquitously expressed Sedlin (TrappC2), and antimorphic or loss of function mutations in *TRAPPC2* cause X-linked spondyloepiphyseal dysplasia Tarda (SEDT) [33]. SEDT results from a chondrocyte-specific defect in the trafficking and secretion of cartilage structural proteins, specifically type II procollagen (Table 2) [34,35]. Moreover, mutations in essential components of COPII-coated vesicles, such as *SEC23A, sec23a* and *sec24d,* which transport secretory proteins from the ER to Golgi, result in cranio-lenticulo-sutural dysplasia (CLSD) [36] and the Zebrafish mutants *crusher* [37] and *bulldog* [38], respectively. In all three conditions, there is accumulation of proteins, in particular fibrillar collagens, within the ER of relevant cell types (Table 2). Finally, loss-of-function mutations in Golgin GMAP-210 (*TRIP11*) causes a lethal skeletal dysplasia in both mice and humans (achondrogenesis type 1A) [39], which is characterized by disrupted Golgi architecture and ER stress due to the intracellular accumulation of perlecan (but not aggrecan or type II collagen) eventually leading to abnormal chondrocyte differentiation and increased apoptosis (Table 2) [39].

In summary, these various genetic studies consistently demonstrate that ‘professionally secreting cells’ such as chondrocytes are highly susceptible to perturbations in ER homeostasis and defects in protein trafficking and secretion. This premise is supported by the observation that deletion or mutation of ubiquitously expressed components of the secretory pathway specifically causes chondrocyte disruption and cartilage defects (Figure 1). Modulation of the secretory pathway in zebrafish mutants by brefeldin A treatment has recently been demonstrated in polycystic kidney disease by Le Corre and colleagues [40], who have proposed that restoration of normal rates of secretory protein synthesis and secretion may be a new target in the treatment of autosomal dominant trafficking defects.

## 5. Incorporation of mutant proteins into the ECM leads to cartilage defects and GSDs

The retention of mutant protein in the ER of cells appears to be a major pathomolecular mechanism underpinning the disease aetiology in a range of GSDs (Figure 1). Therefore, one of the potential targeting avenues suggested in the literature is the use of molecular chaperones, which are small molecules that could potentially aid in the degradation and/or secretion of the mutant proteins into the cartilage ECM [41].

The potential effectiveness of such treatment has been demonstrated for type IV collagen mutations causing hemorrhagic stroke. In a study described by Murray and colleagues [42], two related individuals carrying the same missense mutation presented with differing severity of cerebral haemorrhaging; whilst the parent appeared unaffected, his son showed symptoms of severe porencephaly. In fibroblast cell lines from both individuals, the mutant protein was secreted and incorporated into the ECM of the basement membrane causing morphological abnormalities. However, the cells from the severely affected son secreted less collagen IV and showed an increased retention of the mutant protein in the rER. Treatment with a molecular chaperone (sodium phenyl butyrate) appeared to alleviate the ER stress and restore protein secretion to some extent, thus presenting a promising thera eutic avenue [42].

However, it is important to note that the incorporation of mutant proteins into the ECM may also have deleterious effects and lead to a skeletal condition, as highlighted by the PSACH and MED-causing mutations in the C-terminal domain (CTD) of COMP [19]. In several-documented cases CTD mutant COMP protein is secreted, eliciting either a mild ER stress or no ER stress at all, yet still resulting in dysplasia. The incorporation of mutant COMP into the ECM results in abnormal fibrillar and proteoglycan composition, leading to abnormal chondrocyte clustering, a decrease in proliferation and eventually apoptosis [19]. Moreover, changes in cartilage ECM composition has also been detected in some type II collagenopathies (SEDc and Kniest Dysplasia), diastrophic dysplasia (DTDST) where the cartilage shows signs of under-sulphation of proteoglycans and other ECM abnormalities [43], and finally Schneckenbecken dysplasia where the ECM abnormalities result from a loss-of-function mutation to the SLC35D1 nucleotide-sugar transporter [44]. In all these examples, the ECM defects lead to an altered tissue morphology and cellular organization, thereby affecting the chondrocyte columnar arrangement in the proliferative zone of the growth plate and ultimately impacting on long bone growth.

An in-depth analysis of the structural proteins in the cartilage ECM, in particular the proteins that have a bridging/adaptor function in the ECM, using transgenic mouse models has shown that some of these molecules are actually redundant in cartilage. For example, the genetic deletion of COMP [45], matrilin-3 [46], matrilin-1 [47], type IX collagen [48] and WARP [49] had no effect on gross cartilage structure and long bone growth. Therefore, one can postulate that silencing the defective alleles may pose an exciting therapeutic avenue for skeletal dysplasias resulting from mutations in structural proteins [50]; in fact, Posey and colleagues have tested this approach by using RNA interference [51]. In this particular study, the authors used short hairpin RNA to knock down the expression of both wild-type and mutant COMP, thus reducing the presence of mutant COMP inside and outside the cell [51]. However, this type of approach would not be applicable for the most important structural molecules of cartilage and bone, that is, the collagens and proteoglycans. For example, haplo-insufficiency for type II collagen and aggrecan leads to Stickler syndrome and SED-Kimberley, respectively (Table 3), whereas a lack of perlecan is associated with Schwartz-Jampel syndrome [1]. Interestingly, allele-specific RNA silencing using siRNA has recently been described as potential therapy for Meesmann epithelial corneal dystrophy caused by a point mutation in the keratin gene. Therefore, pending the development of effective methods to deliver such therapies to the dense and largely avascular environment of cartilage, they nonetheless present an exciting future therapeutic avenue.

**Table 3.** **Disease mechanisms in selected GSDs resulting from haploinsufficiency for cartilage structural proteins.**

**Gene**	**Protein**	**Disease**	**In**	**Molecular mechanisms**	**Cell and/or tissue mechanism**
*COL2A1*	Type II collagen	Stickler syndrome type 1	AD	Heterozygous nonsense mutations or out of frame deletions leading to frameshift	Presumed haploinsufficiency for type II collagen Potential antimorpic disruption to collagen fibril structure and cartilage organization
*COL9A1*	Type IX collagen	Stickler syndrome type 4	AR	Homozygous nonsense mutations	Presumed haploinsufficiency for type IX collagen Potential antimorpic disruption to collagen fibril structure and cartilage organization Possible ER stress associated with the degradation of excess collagen α(IX) chains
*COL9A2*		Stickler syndrome type 5		Homozygous for predicted frame shift mutations and a premature termination codon	
*COL9A3*		Stickler syndrome type 6		Homozygous out of frame deletion leading to frameshift and a premature termination codon	
*COL10A1*	Type IX collagen	Metaphyseal chondrodysplasia, Schmid type	AD	Heterozygous nonsense mutations or out of frame deletions leading to frameshift and a premature termination codon	Haploinsufficiency for type X collagen due to NMD of mRNA from mutant allele Possible ER stress associated with NMD Potential disruption to cartilage structural organization
*COL11A1*	Type XI collagen	Fibrochondrogenesis 1	AR	Compound heterozygosity for a loss-of-function mutation and an antimorphic missense mutation (glycine substitution)	Haploinsufficiency of α2(XI) collagen chains due to NMD of mRNA from mutant allele Possible ER stress resulting in a fibroblastic appearance of the chondrocytes Antimorpic disruption to collagen fibril structure producing a fibrous ECM
*COL11A2*		OSMED/WZS	AR	Homozygous for nonsense mutations Absence of α2(XI) collagen chains due to NMD of mRNA from mutant alleles	Potential antimorpic disruption to collagen fibril structure and cartilage organization Possible ER stress associated with the degradation of excess α1(XI) collagen chains
*ACAN*	Aggrecan	Idiopathic short stature	AD	Predicted frame shift mutations leading to a premature termination codon	Presumed haploinsufficiency for aggrecan due to NMD of mRNA from mutant allele Potential antimorpic disruption to cartilage structure and tissue organization
		SED Kimberley	AD		

AD: Autosomal dominant; AR: Autosomal recessive; ER: Endoplasmic reticulum; ECM: Extracellular matrix; GSDs: Genetic skeletal diseases; NMD: Nonsense-mediated degradation.

Mutations that result from aberrant splicing often lead to nonsense-mediated decay of mRNA and associated cell stress or the production of truncated molecules, which may have downstream dramatic effects. This is the case in some cases of Schwartz-Jampel syndrome (SJS) and in dyssegmental dysplasia, Silverman-Handmaker type (DDSH) where an exon skipping mutation leads to a truncated perlecan molecule [2]. However, it is sometimes possible to predict a milder phenotype if the RNA splicing can be partially restored, resulting in a shorter molecule but with a majority of the domains in-frame and correctly folded. Such a rationale was recently applied to the treatment of Duchenne muscular dystrophy by attempting to restore partially functional dystrophin and thus reducing the severity of the disease to a milder Becker muscular dystrophy-like phenotype. Whilst this approach may not be suitable for all cases of GSD, it could be applicable in those diseases where haploinsufficiency and/or retention of the mutant protein leads to a severe phenotype, but a presence of the partially functional mutant molecule in the ECM may lead to a milder condition, and is therefore an interesting therapeutic avenue that could be explored in the future.

## 6. Mechanosensing is important in the pathobiology of GSDs

Primary cilium is an organelle existing on almost every cell type in the human body [52]. In cartilage, cilia have been implicated in important signalling pathways (such as hedgehog and wnt signalling) and have been suggested as a mechanosensory organelle on the cells [53]. Various ECM receptors, including several integrins, are located on or within a close vicinity of the primary cilia and may be important in regulating the cell–matrix interactions [54]. Furthermore, the primary cilia length and prevalence are increased in osteoarthritic cartilage [55] and changes to the cilia organization have been observed in skeletal dysplasias (our unpublished observations) and chondrosarcoma tissues [56].

Disruptions to the columnar organization of proliferative chondrocytes have been described in several mouse models of rare skeletal conditions, in particular those where the ECM structure was affected [57–61]. The change in cell alignment was not dissimilar from abnormalities seen in cartilage-specific integrin knock-out mice, which indicated that the disruption of the cell matrix interactions may in fact be the underlying pathology [62,63]. Moreover, a truncating mutation in integrin α 10 leads to a canine chondrodysplasia [64]. From a therapeutic perspective, it is interesting to speculate that the way a cell senses its environment could be modulated. Indeed, such therapies have in fact been tried in Crohn’s disease where the use of integrin antagonists has shown a dramatic improvement in disease severity [65].

Mechanosensing is important for cartilage development and homeostasis and mutations involving primary cilia molecules have been discovered in several chondrodysplasias to date, including the Verma-Naumoff syndrome, Majewski syndrome, Jeune syndrome, Ellis-van Creveld syndrome, the Sensenbrenner syndrome and Weyers acrofacial dysostosis [1]. In many cases of the skeletal ciliopathies, the underlying disease mechanism is the disruption to the hedgehog-signalling pathway, which is an important pathway regulating cartilage proliferation and differentiation. Interestingly, hedgehog signalling can be partially modulated and/or restored using small molecular treatment, and several reagents including inducible protein reagents based on the Gli1 and Gli3 transcription factors as well as purmorphamine (a small-molecule agonist of Smoothened) are able to activate the Hedgehog pathway and are the proposed reagents currently tested for future treatment of ciliopathies [66].

## 7. Changes to extracellular signalling can also lead to skeletal dysplasia phenotypes

ECM molecules are important for the sequestration and diffusion of endocrine, paracrine and autocrine molecules in the dense cartilage tissue [67]. Mutations inducing changes in the chemical and physical composition of the ECM may therefore have detrimental effects on the signalling pathways driving development and differentiation in cartilage. For example, changes in Indian hedgehog signalling have been detected in DTDST cartilage [68] and in transgenic mice harbouring the deletion of exon 48 in the mouse alpha1(II) procollagen gene [69].

Mutations in the genes encoding molecules important in cartilage signalling have also been implicated in GSDs. Eiken syndrome is a recessive skeletal condition resulting from a truncating mutation in the parathyroid hormone-related peptide type 1 receptor (PTHR1) and mutations in the *PTHR1* have also been found in certain forms of Jansen dysplasia [2]. SHOX haploinsufficiency leads to perturbed programmed cell death of hypertrophic chondrocytes and premature epiphyseal fusion of the distal radius in patients. In the case of truncating and loss of function mutations, silencing of the defective receptors, the use of soluble agonists and activators of the signalling pathways or the use of soluble receptors and enzyme replacement therapy (ERT) can all be proposed as potential therapeutic avenues. For example, in the case of hypophosphatasia due to loss of function mutations in the gene encoding tissue-nonspecific alkaline phosphatase, asfotase alfa (a first-in-class ERT) is undergoing evaluation [70–72].

Conversely, numerous GSDs result from activating mutations affecting the signalling pathways in the cartilage [2]. Mutations within the fibroblast growth factor receptors (FGFR) 1 – 3 cause a wide range of skeletal disorders, including diseases primarily characterized by craniosynostosis such as osteoglophonic dysplasia (*FGFR1*, [73]), Apert (*FGFR2*, [74]), Crouzon (*FGFR2*, [75]), Pfeiffer (*FGFR1* and *FGFR2*, [76]), Beare-Stevenson cutis grata (*FGFR2*, [77]) and Muenke (*FGFR3*, [78]) syndromes, in addition to the dwarfing syndromes ACH, hypochondroplasia and thanatophoric dysplasia (all *FGFR3*, [79]). FGFRs are plasma membrane receptor tyrosine kinases that mediate intracellular signalling upon ligand binding. While the phenotypes of these disorders vary, they all arise as a consequence of constitutive FGFR protein activation, and disruption to downstream signalling cascades.

Other GSDs share this common disease basis with activating mutations found in different genes causing diverse disorders such as Jansen’s metaphyseal chondrodysplasia (*PTH1R*) [80], fibrous dysplasia (*GNAS*, [81]) and brachyolmia type 3 and metatrophic dysplasia (*TRPV4*) (Figure 1) [82]. Similar to *FGFR1 – 3*, *PTH1R* encodes a cell surface receptor (parathyroid hormone/parathyroid hormone-related peptide receptor); however, downstream signalling occurs through several different guanine-nucleotide binding proteins (G-proteins) [83], while *GNAS* encodes the α subunit of one of these G-proteins, Gsα. Constitutive activation of both PTH/PTHrP and Gsα leads to the excessive synthesis of cyclic AMP, disrupted chondrocyte signalling and bone pathology [80,84]. Activating mutations in transient receptor potential vallinoid family member 4 (TRPV4) leads to a delay in bone mineralization and a spectrum of diseases from autosomal dominant brachyolmia to lethal metatrophic dysplasia. *TRPV4* (transient receptor potential vanilloid 4) encodes a calcium permeable cation channel and disease-causing mutations constitutively activate this channel resulting in an uncontrolled influx of calcium into chondrocytes, activation of follistatin [85], increased inhibition of BMP activity [86] and ultimately, improper bone formation (Figure 1). Finally, one of the best examples of this class of GSD is fibrodysplasia ossificans progressive (FOP), which results from heterozygous activating mutations in Activin receptor A, type I/Activin-like kinase 2 (ACVR1/ALK2), a bone morphogenetic protein receptor. The genetic basis of FOP and relevant therapeutic targets and approaches has been extensively reviewed by Frederick Kaplan and colleagues [87]. Briefly, targeting the bone morphogenetic protein signalling pathway has been proposed as a promising therapeutic target and palovarotene, a retinoic acid receptor γ agonist [88], is currently in Phase II clinical trials for patients with FOP.

Existing therapies for GSDs caused by constitutively activating mutations remain limited, although insights into their molecular pathogeneses have yielded some exciting therapeutic targets (Table 4). A murine model introduced with a dominant-negative *FGFR1* construct *in utero* prevented suture fusion within the skull [89], providing proof-of-principle that the abnormal suture fusion of FGFR-related craniosynostosis may be modulated by engineered FGFR receptors. Perhaps more promising as potential therapies, both RNA interference of FGFR2 and pharmacological inhibition of MEK-ERK signalling have been shown to prevent abnormal skeletal phenotypes in a mouse model of craniosynostosis and reduce cranial suture fusion [90,91].

**Table 4.** **Disease mechanisms and potential therapeutic targets in selected GSDs resulting from constitutively activating mutations.**

**Gene**	**Protein**	**Disease**	**In**	**Molecular Mechanisms**	**Cell and/or tissue mechanism**	**Target(s)**	**Treatment**	**Ref.**
*FGFR3*	Fibroblast growth factor receptor 3	Achondroplasia Hypochondroplasia Thanatophoric dysplasia Thanatophoric dysplasia	AD	Missense gain of function missense mutations causing constitutive activation of FGFR3	Reduced chondrocyte proliferation with disrupted growth plate architecture	Pharmacological inhibition of MEK-ERK signalling and modulations of MAPK pathway	BMN111 Statins Meclizine	[92–98]
*PTH1R*	Parathyroid hormone 1 receptor	Metaphyseal chondrodysplasia, Jansen type	AD	Missense mutations causing activation of the cAMP pathway	Reduced chondrocyte proliferation, with premature maturation of chondrocytes and accelerated bone formation	Pharmacological modulations of the PTH-PTHrP receptor pathway	GSK2193874 HC-067047	[80]
*GNAS1*	Guanine nucleotide binding protein, alpha stimulating	Fibrous dysplasia		Activating missense mutations which renders the gene functionally constitutive	Abnormal changes in cell shape and collagen structure	The constitutively active Gsα protein and downstream effectors	Bisphosphonate	[84,103]
*TRPV4*	Transient receptor potential cation channel subfamily V member 4	Brachyolmia type 3 SMD Kozlowski type Metatrophic dysplasia	AD	Missense gain of function mutations causing increased constitutive current before agonist application. Increased intracellular calcium ion concentration and activity	Abnormally thick cartilage with nodular proliferation. Abnormal chondrogenesis and abnormal differentiation of mesenchymal progenitors as well as lack of normal columns of chondrocytes	Blocking the calcium-permeable TRPV4 channel	None tested	[82,85,99–102]
*ACVR1/ALK2*	Activin receptor A, type I/Activin-like kinase 2	Fibrodysplasia ossificans progressiva	AD	Heterozygous activating mutations due to allosteric destabilization of an inactive receptor conformation and therefore a loss of autoinhibition	Formation of a second skeleton of heterotopic bone including congenital malformations of the great toes and progressive heterotopic endochondral ossification	BMP signalling pathway:- Blocking activity of the mutant receptor Blocking inflammatory Triggers Blocking Progenitor Cells	Palovarotene in Phase II clinical trails	[87,88]

AD: Autosomal dominant; AR: Autosomal recessive; BMP: Bone morphogenetic protein; GSDs: Genetic skeletal diseases; SMD: Spondylometaphyseal dysplasia.

A significant development in efforts to treat ACH was the discovery that CNP overexpression rescues the phenotype in an ACH mouse model by inhibiting FGFR3 downstream signalling through the MAPK pathway [92]. Recent efforts have focused upon optimizing the pharmacological properties of CNP by designing and validating CNP analogues such as BMN 111 [93,94], or improving production yields [95]. An alternative to CNP-based therapy has involved the repositioned use of a FDA-approved drug (meclizine used for treatment of motion sickness) [96]. In this study, human chondrosarcoma cells expressing constitutively active mutants of *FGFR3* that cause thanatophoric dysplasia, SADDAN and achondrogenesis were treated *in vitro* with meclozine and this ameliorated abnormally suppressed cell proliferation. Comparison with the effect of CNP on the same cell models revealed that meclozine was as efficient as CNP in attenuating the abnormal FGFR3 signalling. Subsequent studies validated this result in a mouse model of ACH, showing that the bone lengths of both wild-type and mutant mice treated with meclozine were significantly longer than in untreated mice [97]. Given that meclozine is already an FDA-approved drug, it is ideally positioned for assessment for clinical use in the treatment of short stature in achondrogenesis.

The use of statins to treat FGFR3-related disorders, ACH and tanatophoric dysplasia type I (TD1) has recently been proposed [98]. Yamashita *et al*. have demonstrated that statins, previously used in the treatment of tumours and of disorders in the cardiovascular, nervous and immune systems, were able to upregulate the expression of sox9, type II collagen and aggrecan in cell and animal models of ACH and TD1 [98]. They have also demonstrated a decrease in the protein levels of FGFR3 in the patient-derived induced pluripotent stem cells (iPSCs) differentiated into the chondrogenic lineage and in the Fgfr3^Ach^ mouse model of ACH. Interestingly, the decrease in the protein level did not correlate with a decrease in mRNA level for FRFR3, which was actually slightly increased upon the statin treatment. Treatment of chondrocytes derived from the Fgfr3^Ach^ mice with lovastatin in the presence of proteasomal inhibitors revealed that the statin partially upregulated the proteosomal degradation of FGFR3 in the treated cells and animals [98].

Several approaches have been proposed as possible therapeutic interventions for TRPV4-related disease [99]. Existing agents capable of blocking the calcium-permeable TRPV4 channel (GSK2193874 [100] and HC-067047 [101]) represent candidate drugs that would be highly appropriate for administration to mouse models of TRPV4-related disease [102] and subsequent phenotypic analysis. Alternatively, inhibition of TRPV4 protein expression using shRNA might be another way to circumvent the harmful effect of uncontrolled calcium influx into chondrocytes.

Perhaps the most effective clinically approved treatment for a GSD caused by a constitutively activating mutation is the intravenous administration of bisphosphonates to treat fibrous dysplasia. This class of drugs possesses a common basic structure similar to pyrophosphate that inhibits bone resorption and has been used with success for decades. In addition to a decreased turnover of bone, the radiological aspect of existing bone lesions is improved, as is the experience of pain reported by patients [103]; however, a subset of patients remain nonresponsive to bisphosphonate treatment. The relatively recent discovery of GNAS as the disease locus of fibrous dysplasia raises the possibility of targeting the constitutively active Gsα protein and downstream effectors in future therapeutic interventions.

## 8. Reduced chondrocyte proliferation and increased dysregulated apoptosis are the downstream effects and a shared disease mechanism of many GSD mutations

Several studies have recently demonstrated that reduced chondrocyte proliferation, increased and/or dysregulated apoptosis in the growth plates of mouse models is a major pathological component of various GSDs, including those resulting from mutations in genes encoding cartilage structural proteins (*Comp, Matn3* and *Col2a1*) [8,12,104], a sulphate transporter (*Slc26a2*) [105] and components of the trans-golgi network (GMAP-210) [32]. These pathomolecular mechanisms are particularly relevant to those GSDs that have a significant epiphyseal involvement, such as PSACH-MED, DTDST and the type II collagenopathies, but are perhaps not so relevant for metaphyseal chondrodysplasias such at MCDS where the pathology involves only non-proliferating hypertrophic chondrocytes [16].

Defining the relative contribution of reduced chondrocyte proliferation, increased and/or dysregulated apoptosis to growth plate dysplasia and reduced bone growth is experimentally challenging; however, the study of novel ‘ER-stress phenocopies’ has recently provided new insight into the specific impact of these different disease mechanisms [16,106,107]. The cartilage-specific expression of mutant forms of thyroglobulin has confirmed that reduced chondrocyte proliferation resulting from an intracellular stress caused by the accumulation of a misfolded protein and in the absence of perturbations to apoptosis was sufficient to cause a significant reduction in long bone growth [107].

In summary, these innovative studies therefore defined reduced chondrocyte proliferation as a major determinant of reduced bone growth in epiphyseal dysplasias, which holds the promise of therapeutic intervention or as a robust readout of drug efficacy in these pre-clinical models of GSDs.

## 9. Soft tissue complications are a common factor in GSDs

Cartilage, bone, tendon, ligament and skeletal muscle are all tissues of the mesenchymal lineage. It is therefore not surprising that many of the structural molecules implicated in GSDs are also expressed in more than one of these tissues. Musculoskeletal complications are therefore an additional complication that is important to consider in terms of clinical management of GSDs. Patients with MED [108,109], Camurati-Englemann disease [110,111], Marfan syndrome [112,113] and Schwartz-Jampel syndrome [114,115] present with musculoskeletal complications (muscle weakness, muscle stiffness, joint laxity, easy fatigue) that are often difficult to diagnose upon biopsy alone. Understanding the disease mechanisms underlying these clinical complications is of utmost importance, especially considering that these symptoms can manifest prior to the onset of skeletal manifestations and can lead to an initial misdiagnosis. Moreover, mesenchymal tissues are highly mechanically responsive and able to regulate gene expression depending upon the mechanical stimulus [116,117]. It is therefore interesting to speculate that the understanding of the musculoskeletal complications associated with GSDs may lead to a better physiotherapy regime and management of the patients in the future.

## 10. Biomarkers are important for monitoring disease progression and efficacy of treatment

A lack of relevant disease biomarkers has been a key factor to the delay in the development of new therapeutic targets, largely due the interdependency between biomarker and drug development pipelines [118]. Worryingly there are currently no reliable and readily quantifiable biomarkers that allow pre-symptomatic diagnosis of most GSDs, or to monitor responses to therapeutic regimes. This lack of suitable specific biomarkers reflects the complexity of the biomarker pipeline, which involves the co-dependent processes of identification, verification, validation and reliable detection and quantification in easily obtained biological samples such as blood, urine and cell culture medium.

The complete ECM portrait of bone and cartilage comprises a limited number of ∼300 different components termed the ‘core matrisome’ [118], which limits the available ‘pool’ of potential proteins and/or degradation products that may be found in serum. The determination of biomarkers in serum or urine is widely used to screen for specific pathologies of various organs. For example, individuals with skeletal disorders can be monitored for biomarkers of bone formation, bone resorption or cartilage degradation, which helps to discriminate between different types of skeletal pathologies. Moreover, the information obtained from this analysis can be relevant for individual treatment regimes, for example, not every patient displaying reduced bone mass will necessarily profit from the commonly used anti-resorptive medication. In addition to the well-established relevance of biomarkers in disease diagnosis and personalized treatment, their altered concentrations can also be causative for disease-associated pathologies, such as the case for pyridoxal-5′-phosphate and pyrophosphate in hypophosphatasia, TGFß in geleophysic dysplasia or for FGF23 in hypophosphatemic rickets. In particular, the latter example underscores the relevance of identifying disease-associated biomarkers since FGF23 is now considered to be one of the key regulators of phosphate homeostasis in humans [118].

The use of Omics technologies and Systems analysis to identify phenotype and/or genotype-specific disease profiles promises to provide a plethora of novel putative biomarkers that can be fully investigated through relevant cell and animal models. Ultimately, personalized treatments and care strategies will require relevant biomarkers to monitor efficacy of treatment and disease progression. Therefore, novel biomarkers are urgently required for detecting pre-clinical disease, to monitor disease progression and as a prerequisite for clinical trials of new therapeutic targets. This lack of suitable biomarkers is recognized as a major hindrance in translating research into patient benefits.

## 11. European-wide networks generate critical mass for diagnostic and research excellence: the search for common therapeutic targets

In Europe, the skeletal genetics field has been fortunate over the last 15 years to secure significant funding from the European Commission, via its various Framework Programs, to establish three contiguous clinical and/or research networks focused on rare skeletal diseases. The origin of these successful large-scale collaborative projects lies with a Concerted Action (1995 – 2000), which provided the first opportunity for both clinical and research experts in GSDs to interact in a Pan-European environment and this ultimately fostered important collaborations and ground-breaking ideas that would lead to future GSD networks.

The FP5-funded European Skeletal Dysplasia Network (ESDN) for research and diagnosis (2002 – 2006) was one of the first networks of expertise in the field of rare diseases to use information and communications technology tools for the purposes of tele-expertise and medical diagnosis. Since September 2003, ESDN has received over 2000 referrals through an on-line Case Manager and 450 users have accessed ESDN from 45 different countries worldwide. During this initial funding period, research activities within ESDN developed several relevant mouse models of GSDs and identified the first disease mechanisms, which would later be defined as hallmark features of disease pathology and be tentatively proposed as potential therapeutic targets.

EuroGrow (2007 – 2010) was an FP6 project that focused on the phenotyping of mouse models of a select group of GSDs in order to develop and validate experimental approaches for deep-phenotyping including omics-based analysis. This project successfully identified a range of common disease mechanisms between phenotypically different GSDs and laid the foundation for future large-scale projects.

The FP7-funded SYBIL project (Systems Biology for the functional validation of genetic determinants of skeletal diseases; http://www.sybil-fp7.eu) is a large-scale collaborative project that aims to functionally validate genetic determinants of common and rare skeletal diseases to gain a mechanistic understanding of the disease processes and age-related changes and to deliver new and validated therapeutic targets. The outcomes of this project will include the generation and deep phenotyping of a diverse range of cell and animal models of GSDs that will generate new knowledge on disease mechanisms. The major strength of this coordinated and multidisciplinary approach is the use of System Biology to underpin the extensive ‘omics’-based analysis that will identify common disease mechanisms and potential therapeutic targets in an unbiased and iterative process.

Finally, National GSD networks such as the Skeletal Dysplasia Group (UK), SKELNET (Germany) and Skeldys.org (Switzerland) have provided a combination of both formal and informal discussion and diagnostic forums, whilst the establishment within several EU countries of National Centers of Excellence for specific GSDs has generated critical mass for delivering clinical best practice and translational research.

In summary, trans-European networks of clinical and research excellence in GSDs have a proven track record in delivering an efficient world class diagnostic service and generating new knowledge on disease mechanisms that will translate into novel therapeutic targets that might even be shared amongst groups of different disease (i.e. Common amongst the Rare). Moreover, interactions with patient self-support groups, the International Rare Diseases Research Consortium (IRDiRC) and the establishment of a European Reference Network in the GSD domain will all help to accelerate and deliver translational research through broad international collaborations and the sharing of best practice.

## 12. Clinical utility and patient expectations

The development of potential therapeutic targets to the point at which clinical trials can be performed and pharmacological agents ultimately brought to market is a notoriously challenging process with high attrition rates [119]. The low prevalence of individual GSDs is a further challenge to the viable commercial development of products in this area. In some cases, it has been possible to apply drugs already used for the treatment of related conditions to rare GSDs. For example, the use of bisphosophonates in the treatment of adults and particularly children with Osteogenesis Imperfecta (OI) has been credited with reducing fracture frequency and improving quality of life [120,121]; however, recent studies have called this into question [122]. The example of the bisphosphonates illustrates some of the challenges of demonstrating clinical utility in the use of drugs in this group of diseases. This is even more difficult in conditions where, unlike OI, the relatively hard end points of fracture frequency and changes in vertebral morphology are not applicable. It has been suggested that changes in final adult height might be a relevant measure. It is unlikely that changes of sufficient magnitude to truly alter functional outcome will be achieved in many of the GSDs, particularly when the condition is associated with very significant reductions not only in stature but also in reach. Alternative measures of improved symptom control, particularly reduction in pain and neurological symptoms with improvements in mobility, will need to be assessed. A particular challenge in this area will be the lack of availability of baseline data from which assessments of change can be made, even for the most common of the GSDs.

Recent discussions with support groups around the clinical trials currently being carried out for the BioMarin product BMN-111 have emphasized the importance of these issues. Concerns have been expressed that undue focus on stature is inappropriate in the context of the symptoms people with ACH have on a daily basis, whilst a reduction of the neurological impairment associated with neural axis compression would be extremely welcome.

The rarity of the GSDs will pose significant challenges not only to those developing therapeutic agents but also to regulators and healthcare commissioners and providers. The relatively small market available for drugs targeted at specific molecular targets or even specific genetic pathways limits the financial viability of such agents and is part of the wider debate around the development of treatments for rare diseases [123].

## 13. Expert opinion

The extensive clinical variability and genetic heterogeneity of GSDs, coupled with complex disease mechanisms, renders this extensive group of rare diseases a bench to bedside challenge. Indeed, this large number of different and highly complex phenotypes makes the identification, validation and development of potential therapies almost impossible for anything other than the most common GSDs. As an alternative approach, we might consider identifying genotype- and/or phenotype-independent ‘core disease mechanisms’ that are shared amongst families of clinically unrelated GSDs. This approach would allow the focusing of resources into several areas of concerted investigation that have the potential to identify and validate therapeutic targets with a broad application to GSDs, inherited connective tissues as a whole and rare genetic disease in general. Indeed, Jürgen Spranger first suggested the idea of ‘bone dysplasia families’ in 1985 [124] and proposed that phenotypes with a similar clinical and radiographic phenotype would likely have a similar disease mechanism. Thirty years later, we can now expand upon this pioneering concept and propose that common disease mechanisms can also be shared amongst clinically different phenotypes (‘common amongst the rare’).

In this context, ER stress has been associated with a diverse range of genetic diseases and chronic conditions such as skeletal dysplasia (as discussed in this review), myopathy [125], cerebro-vascular [42], kidney [126], ischaemia and cardiovascular diseases [127]. Moreover, ER stress is emerging as a very attractive target that is being successfully exploited in a broad range of diseases including neuropathy, juvenile-onset open-angle glaucoma, obesity, diabetes, asthma and epidermolysis bullosa, to name but a few. Historically many GSDs were considered diseases of the ECM and proposed therapeutic interventions involved the removal and/or correction of the relevant mutated gene or abnormal gene product. This was particularly the case with dominant-negative mutations in the large structural proteins of the cartilage ECM such as type II collagen [50]. However, emerging knowledge suggests that the primary genetic defect may be less important than the cells’ response to the expression of the mutant gene product [107]. Moreover, the largely overlooked response of a cell (i.e. chondrocyte) to the abnormal extracellular environment is also important for disease progression as illustrated by several GSDs discussed in this review.

It is important that ‘omics’-based approaches and technologies are systematically applied to the study of rare GSDs so that definitive reference profiles and disease signatures are generated for each phenotype. These can then be used in a Systems Biology approach to identify both common and dissimilar pathological signatures and disease mechanisms. This approach is entirely dependent upon relevant *in vitro* and *in vivo* models (and also novel ‘disease-mechanism phenocopies’ [107]) for testing new diagnostic and prognostic tools and for determining the molecular mechanisms that underpin the pathophysiology so that effective therapeutic treatments can be developed and validated. This approach will eventually lead to personalized treatments and care strategies centred on shared disease mechanisms with the use of relevant biomarkers to monitor the efficacy of treatment and disease progression.

It is vital that all relevant stakeholders are involved from the outset in defining the appropriate outcomes of any potential therapeutic regime. The perceptions of a successful therapy can differ widely between the clinical academic community and the relevant patient-support groups and it is vital that there is engagement on all these issues.

In summary, the identification of causative genes and mutations for GSDs over the last 20 years, coupled with the generation and in-depth analysis of a plethora of relevant cell and mouse models, has derived new knowledge on disease mechanisms and suggested potential therapeutic targets. The fast-evolving hypothesis that clinically disparate diseases can share common disease mechanisms is a powerful concept that will generate critical mass for the identification and validation of novel therapeutic targets and biomarkers.

**Article highlights.**

- Endoplasmic reticulum stress is a shared disease mechanism and potential therapeutic target in a diverse range of Genetic skeletal diseases (GSDs) resulting from dominant-negative mutations in cartilage structural proteins.
- Disruption to protein trafficking in chondrocytes leads to a variety of chondrodysplasia phenotypes, which highlights that ‘professionally secreting cells’ such as chondrocytes are highly susceptible to perturbations in ER homeostasis and defects in protein trafficking and secretion.
- The incorporation of mutant proteins into the extracellular matrix leads to changes in the composition and properties of cartilage.
- Reduced chondrocyte proliferation, increased and/or dysregulated apoptosis are common downstream effects for a range of different GDS and are robust readouts for pre-clinical studies.
- Numerous GSDs result from mutations affecting signalling pathways in cartilage and these are the targets of new therapeutic interventions.

This box summarizes key points contained in the article.
